# Genome-wide identification and characterization of perirenal adipose tissue microRNAs in rabbits fed a high-fat diet

**DOI:** 10.1042/BSR20204297

**Published:** 2021-04-27

**Authors:** Jie Wang, Jiahao Shao, Yanhong Li, Mauricio A. Elzo, Xianbo Jia, Songjia Lai

**Affiliations:** 1 College of Animal Science and Technology, Sichuan Agricultural University, Chengdu 611130, China; 2 Department of Animal Science, University of Florida, Gainesville, FL 32611, U.S.A

**Keywords:** Adipogenesis, High-fat diet, MiRNAs, Rabbits

## Abstract

MicroRNAs (miRNAs) are a class of endogenous single-stranded RNA molecules that play an important role in gene regulation in animals by pairing with target gene mRNA. Extensive evidence shows that miRNAs are key players in metabolic regulation and the development of obesity. However, the systemic understanding of miRNAs in the adipogenesis of obese rabbits need further investigation. Here, seven small RNA libraries from rabbits fed either a standard normal diet (SND; *n*=3) or high-fat diet (HFD; *n*=4) were constructed and sequenced. Differentially expressed (DE) miRNAs were identified using the edgeR data analysis package from R. Software miRanda and RNAhybrid were used to predict the target genes of miRNAs. To further explore the functions of DE miRNAs, Gene Ontology (GO) and Kyoto Encyclopedia of Genes and Genomes (KEGG) pathway enrichment analysis were performed. A total of 81449996 clean reads were obtained from the seven libraries, of which, 52 known DE miRNAs (24 up-regulated, 28 down-regulated) and 31 novel DE miRNAs (14 up-regulated, 17 down-regulated) were identified. GO enrichment analysis revealed that the DE miRNAs target genes were involved in intermediate filament cytoskeleton organization, intermediate filament-based process, and α-tubulin binding. DE miRNAs were involved in p53 signaling, linoleic acid metabolism, and other adipogenesis-related KEGG pathways. Our study further elucidates the possible functions of DE miRNAs in rabbit adipogenesis, contributing to the understanding of rabbit obesity.

## Background

Obesity is spreading rapidly in both developed and developing countries, becoming a considerable public health concern threatening human health [1]. According to the definition of obesity (body mass index (BMI) higher than 30 kg/m^2^) by the World Health Organization (WHO), females suffer a higher incidence of obesity than males, and this phenomenon tends to occur at a younger age [2,3]. The global epidemic of obesity stems from the increased adipose mass that results from an imbalance between dietary energy intake and consumption [4]. White adipose tissue (WAT) is widely distributed in subcutaneous tissues and around internal organs, which play a wide range of functions in metabolic regulation and physiological homeostasis, far beyond the simple model of fat storage [5]. Excessive accumulation of WAT is considered to be a risk factor for morbid obesity and some obesity-related diseases [6,7].

The underlying mechanism of obesity is a multifactorial pathological process that involves the interaction between genes and environment [8]. MicroRNAs (miRNAs) are a class of small RNA molecules that play a gene-regulatory role at the post-transcriptional level [9]. In recent years, miRNAs regulation has been confirmed to be involved in fat metabolism and the development of adipose tissue. For example, miR-125a-5p promotes proliferation and inhibits differentiation of adipocytes by regulating *Kruppel-like factor 13* (*KLF13*) [10]. *PPARγ*, *C/EBPα*, and *FABP4* are crucial transcription factors in the development and function of the adipose tissue and markers of lipogenesis [11]. MiR-148a-3p significantly up-regulates the expression levels of *PPARγ*, *C/EBPα*, and *FABP4*, thereby promoting intracellular triglyceride accumulation [12]. Moreover, studies in animals model and humans also showed that many miRNAs play positive or negative functions in adipose tissue, including regulating the differentiation of adipocyte, metabolic, and endocrine functions [13,14]. Diet-induced fat in rabbits has a similar lipid metabolism to humans, making rabbits a reliable and low-cost obesity research model [15]. However, the molecular mechanisms of adipogenesis by mediating miRNAs transcriptomes in high-fat diet (HFD)-induced obese rabbits requires further research.

The objective of the present study was to investigate the role of miRNAs in adipogenesis by sequencing and analyzing perirenal adipose tissue miRNAs from rabbits fed a standard normal diet (SND) or HFD to obtain new insights into miRNAs’ regulatory functions and contribute to the understanding of epigenetic mechanisms influencing fat metabolism in obese rabbits.

## Materials and methods

### Animals and ethics statement

The animals in the present study were 24 female Tianfu black rabbits from a strain bred at the Sichuan Agricultural University in China that were 35 days of age. Rabbits were randomly divided into two groups and fed either a SND or HFD (10% lard was added to the SND) for 4 weeks. Animals had free access to water and were fed twice a day. Each rabbit was housed in a clean iron cage (600 × 600 × 500 mm) and kept in an environmentally controlled room. At the end of the study, weak (rabbits kept away from the feed and did not eat continuously for more than 2 days), disabled (rabbits with obvious limb deformities), and sick (rabbits with significant skin diseases) rabbits were eliminated. Rabbits were classified as obese using the method described in our previous study [16]. Briefly, body weight, body weight gain, and BMI were measured as markers of obesity. Serum TG concentration and body fat rate were also measured as the markers. Four HFD rabbits meeting the obesity requirements and three SND rabbits were used for sampling. All experiments in the present study involving animals were performed under the direction of the Institutional Animal Care and Use Committee from the College of Animal Science and Technology, Sichuan Agricultural University, China (DKY-B2019202015).

### Samples collection and blood measurement

Four HFD rabbits and three SND rabbits that passed the screening process were fasted overnight and humanely killed by electric shock then bloodletting from the jugular vein for sampling. Briefly, blood samples from rabbits were collected the next morning using vacutainer tubes. Then, perirenal adipose tissue samples were taken immediately on the ice. Tissue blocks were placed in 4-ml eppendorf (EP) tubes and stored in a −80°C freezer. Blood samples were centrifuged at 4°C for 5 min and the serum was transferred to clean frozen pipes and stored at −80°C. Serum triglyceride concentrations were obtained by personnel of the Lilai Biological Company (Lilai, Chengdu, China). Briefly, serum biochemical indicators were measured using an enzyme-linked immunosorbent assay (ELISA) with a commercially available ELISA kit (Jianglai, Shanghai, China) on a microplate reader adapted for rabbits (MK3, Thermo Fisher Scientific Shanghai Instruments Co Ltd, Shanghai, China) following the manufacturer’s protocol.

### RNA extraction and quantitative real-time PCR

Total RNA from perirenal adipose tissue samples was extracted using RNAiso Plus Reagent (Invitrogen, Hong Kong, China), following the guidelines of the manufacturer. Subsequently, the purity, concentration, and integrity of RNA were determined by Agilent 2100 Bioanalyzer system (Agilent Technologies, Carlsbad, CA, U.S.A.) and Qubit 2.0 fluorometer (Life Technologies, Carlsbad, CA, U.S.A.), and only RNA meeting quality criteria (A_260_/A_280_ = 1.6–1.8; concentration ≥ 200 ng/µl; RNA integrity number > 7) was used for the trial. Reverse transcription of miRNAs was performed using the Mir-X™ miRNA First-Strand Synthesis Kit (TakaRa, Dalian, China), following the manufacturer’s protocol. Quantitative real-time PCR (qRT-PCR) was performed in triplicate using the TB Green™ Premix Ex Taq™ II (TakaRa, Dalian, China) on a CFX96 instrument (Bio-Rad, U.S.A.), and relative expression levels of miRNAs were calculated by 2^−ΔΔ*C*_T_^ method.

### Small RNA library construction and sequencing

To identify the changes of miRNAs in perirenal adipose tissue, we constructed seven small RNA libraries (SND-1, SND-2, SND-3, HFD-1, HFD-2, HFD-3, HFD-4) from the SND and HFD rabbits. The small RNA libraries were constructed using TruSeq Small RNA Sample Prep Kits (Illumina, San Diego, U.S.A.), following the manufacturer’s instructions. Small RNA of sizes ranging from 15 to 30 nt were isolated using a 15% PAGE gel. The 5′ and 3′ adaptors were ligated sequentially to the small RNA using a T4 RNA ligase (Promega, U.S.A.) and then amplified by qRT-PCR. Lastly, the libraries were sequenced using the Illumina HiSeq 2500 platform (Illumina, San Diego, U.S.A.), and then 50 bp paired-end reads were generated.

### Identification of miRNAs

SOAPnuke software (https://github.com/BGI-flexlab/SOAPnuke) was used to filter the sequencing reads, and after removing the 3′ containing sequencing adaptors, reads larger than 18 nt (clean reads) were retained [17]. The miRBase database (http://www.mirbase.org) was used as a reference database, and software package miRDeep2 2.0.0.8 (https://github.com/rajewsky-lab/mirdeep2) was used to identify novel miRNAs from unannotated reads [18]. The mapped sequences contained some known types of RNA (tRNA, rRNA, snRNA, snoRNA), repeated sequences, and mRNA degraded fragments. Small RNA reads of 18 nt or greater were aligned to rabbit reference genomes with Bowtie2 (http://bowtie-bio.sourceforge.net/bowtie2/manual.shtml) and compared with Rfam database (http://rfam.xfam.org), genebank database (https://www.ncbi.nlm.nih.gov/genbank/), and Repbase database (https://www.girinst.org/repbase) to remove known types of RNA sequences and repeated sequences [19–21]. Next, the filtered small RNA reads were compared with the miRbase database to identify known miRNAs that could be directly used for subsequent analyses. The edgeR data analysis package from R was used for differential expression analysis. The threshold was defined to be |log2(fold change)| ≥ 1 and *P*<0.05.

### Target gene prediction and functional enrichment analysis

Software miRanda (http://www.microrna.org/microrna/home.do) and RNAhybrid (https://bibiserv.cebitec.uni-bielefeld.de/rnahybrid/) were used to predict the target genes of miRNAs, and the intersection of the predicted results was taken as the outcome [22]. Online software DAVID Bioinformatics Resources 6.7 (https://david.ncifcrf.gov/home.jsp) was used to perform Gene Ontology (GO) and Kyoto Encyclopedia of Genes and Genomes (KEGG) pathway enrichment analysis [23]. Differences were considered to be statistically significant at *P*<0.05.

## Results

### Establishment of the obese rabbit model

**Figure 1:**
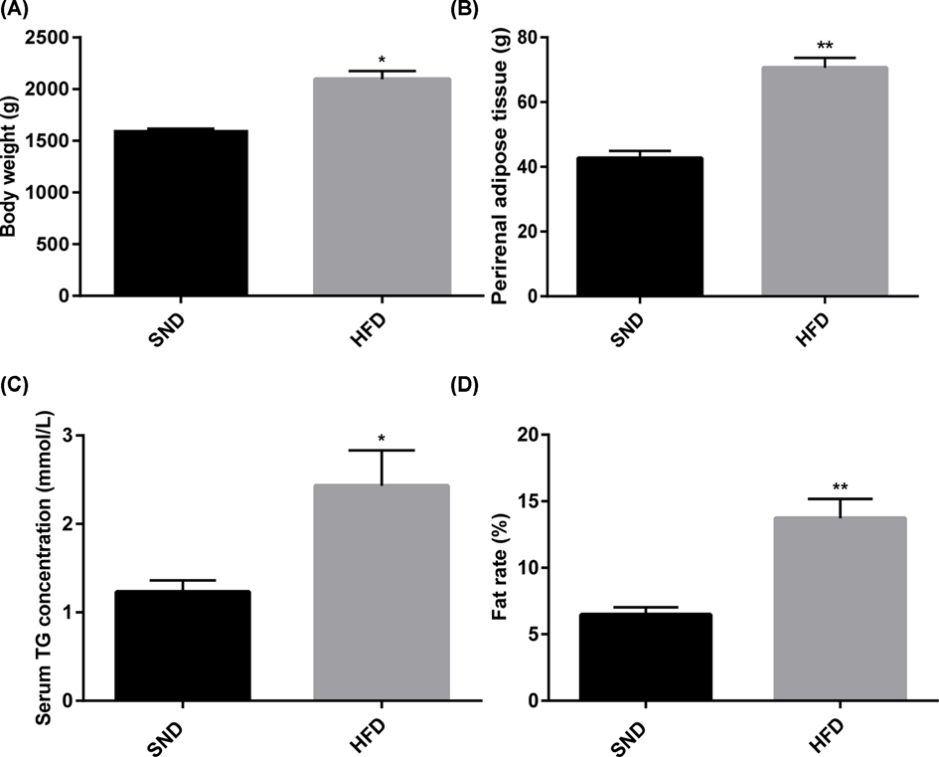
Establishment of the obese rabbit model Body weight (**A**), weight of perirenal adipose tissue (**B**), serum TG concentration (**C**), and fat rate (**D**) in rabbits fed either a SND or HFD for 4 weeks. Data are expressed as means ± SEM; *n*=3 for SND group and *n*=4 for HFD group; *P<0.05; **P<0.001.

In our study, HFD rabbits had higher body weight after 4 weeks of HFD treatment (Figure 1A). The total weight of perirenal adipose tissue was heavier in the HFD rabbits than in the SND rabbits (Figure 1B). Moreover, serum triglyceride (TG) concentration and body fat rate were also used as markers of obesity. When comparing the above indicators in the HFD and SND rabbits, serum TG concentration and body fat rate were consistently and markedly up-regulated (Figure 1C,D). Thus, we concluded that HFD rabbits achieved experimental obesity and could be used for further trial.

### Quality assessment of small RNA sequencing

After raw reads were quality filtered, a total of 81449996 clean reads were obtained from the seven small RNA sequencing libraries. The Q20 (percentage of reads with a Phred quality value > 20) ranged from 99.54 to 99.86%, and the GC content of libraries ranged from 45.74 to 47.72%. The clean reads were mapped to the rabbit reference genome, and the mapped rate ranged from 84.44 to 90.69% (Supplementary Table S1). Further, the average length of most reads was 22 nt in the seven small RNA libraries, which was consistent with the length characteristics of animal miRNAs (Supplementary Table S2).

### Screening of known and novel miRNAs

Mapped sequences were compared with the Rfam, genebank, and Repbase databases for classification and annotation. The number of high-quality miRNA sequences obtained for each sample were 7383340 for SND-1, 6678939 for SND-2, 10137372 for SND-3, 6492570 for HFD-1, 6984965 for HFD-2, 7292413 for HFD-3, and 7475155 for HFD-4 (Table 1). Next, the filtered small RNA reads were perfectly compared with mature rabbit miRNAs in the miRbase database to identify 671 known miRNAs (Supplementary Table S3). Lastly, 488 novel miRNAs were identified using software package miRDeep2 (Supplementary Table S4).

**Table 1:** Distribution of total sRNA identified in the SND and HFD rabbits^1^

	SND			HFD			
	SND-1	SND-2	SND-3	HFD-1	HFD-2	HFD-3	HFD-4
Mapped sequences	8900721	8481469	15537856	8730388	8904422	10084871	10171314
miRNA	7383340	6678939	10137372	6492570	6984965	7292413	7475155
Precursor	1036389	937443	554794	836396	644445	1017783	1081981
rRNA	104255	79579	100251	75058	79824	122625	116755
sRNA	81618	214085	131953	126184	118746	117323	134138
snRNA	501	551	1073	481	570	532	556
snoRNA	72246	31235	54692	23323	28657	34689	45511
tRNA	7796	3150	1422	6626	2771	3269	8992
Unannot	211560	146655	470768	200255	193311	289598	397779
Unmap	332442	496160	839678	415599	477062	528274	440256

1 HFD, high-fat diet; SND, standard normal diet.

### Screening of differentially expressed miRNAs

**Figure 2:**
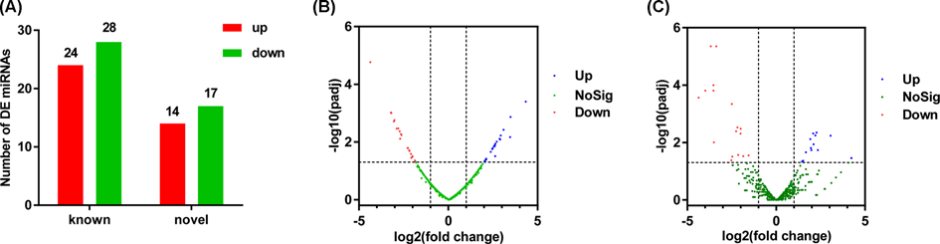
Screening of DE miRNAs (**A**) The number of known and novel DE miRNAs. Volcano map of known (**B**) and novel (**C**) DE miRNAs screened between the SND (*n*=3) and HFD (*n*=4) groups and was built based on log2(fold change) and −log10(*P*_adj_).

To explore the expression patterns of all known and novel miRNAs in the SND and HFD rabbits perirenal adipose tissue, differential analysis of the miRNAs was performed using EdgeR. A total of 52 known differentially expressed (DE) miRNAs (24 up-regulated, 28 down-regulated) and 31 novel DE miRNAs (14 up-regulated, 17 down-regulated) were identified in the SND and HFD rabbits, respectively (Figure 2A). Values of log2(fold change) and −log10(*P*_adj_) were used to construct volcano figures for known (Figure 2B) and novel (Figure 2C) DE miRNAs.

### Enrichment analysis of known DE miRNAs target genes

**Figure 3:**
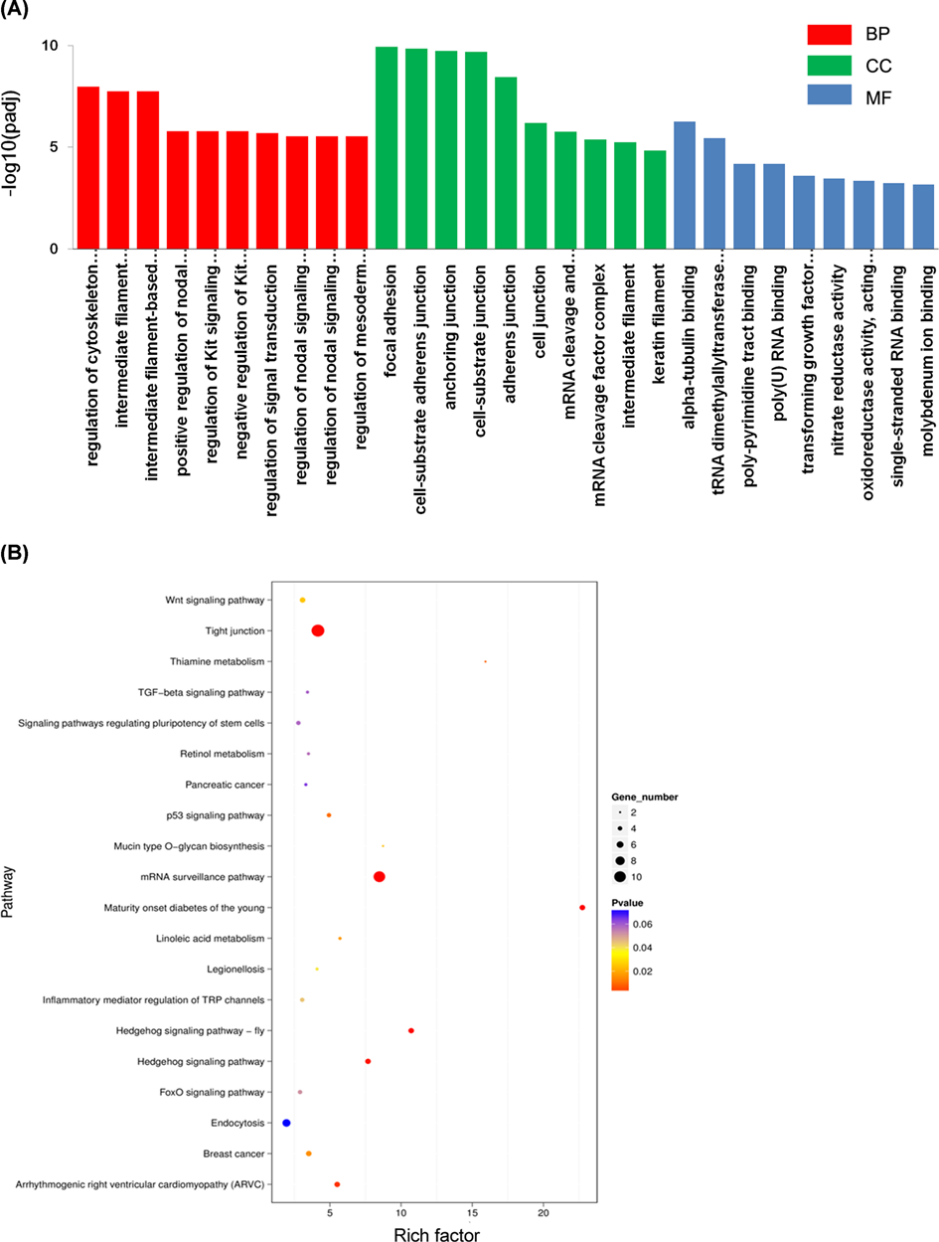
Enrichment analysis of known DE miRNAs target genes (**A**) The results from known DE miRNAs target genes were applied to GO analysis, with only showing the top ten significantly enriched GO terms in BP, CC, and MF (*P*<0.05). (**B**) KEGG pathway analysis of known DE miRNAs target genes, with only showing the top 20 significantly enriched pathways (*P*<0.05). Rich factor = (target genes annotation in term/target genes with KEGG annotation)/(genes annotation in term/all genes with KEGG annotation).

To better study the biological functions of the DE known miRNAs, we used software to obtain 190 known DE miRNAs target genes. GO analysis of known DE miRNAs target genes found a total of 1232 enriched GO terms (885 biological processes (BP), 164 cellular components (CC), and 183 molecular functions (MF)) (Table S5). Multiple significance tests showed that 371 out of 1232 GO terms (30.11%) were significantly enriched (*P*<0.05). The main BPs involved in known DE miRNAs target genes included focal adhesion, α-tubulin binding, and regulation of cytoskeleton organization. The top ten significantly enriched terms in the BP, CC, and MF categories are shown in Figure 3A. The KEGG pathway analysis showed that known DE miRNAs target genes were enriched in 123 pathways including pathways for mRNA surveillance and maturity-onset diabetes of the young and that 14 pathways (11.38%) were significantly enriched (Supplementary Table S6). Besides, a scatter analysis was carried out for the first 20 pathways to intuitively show the significance of these pathways (Figure 3B).

### Enrichment analysis of novel DE miRNAs target genes

**Figure 4:**
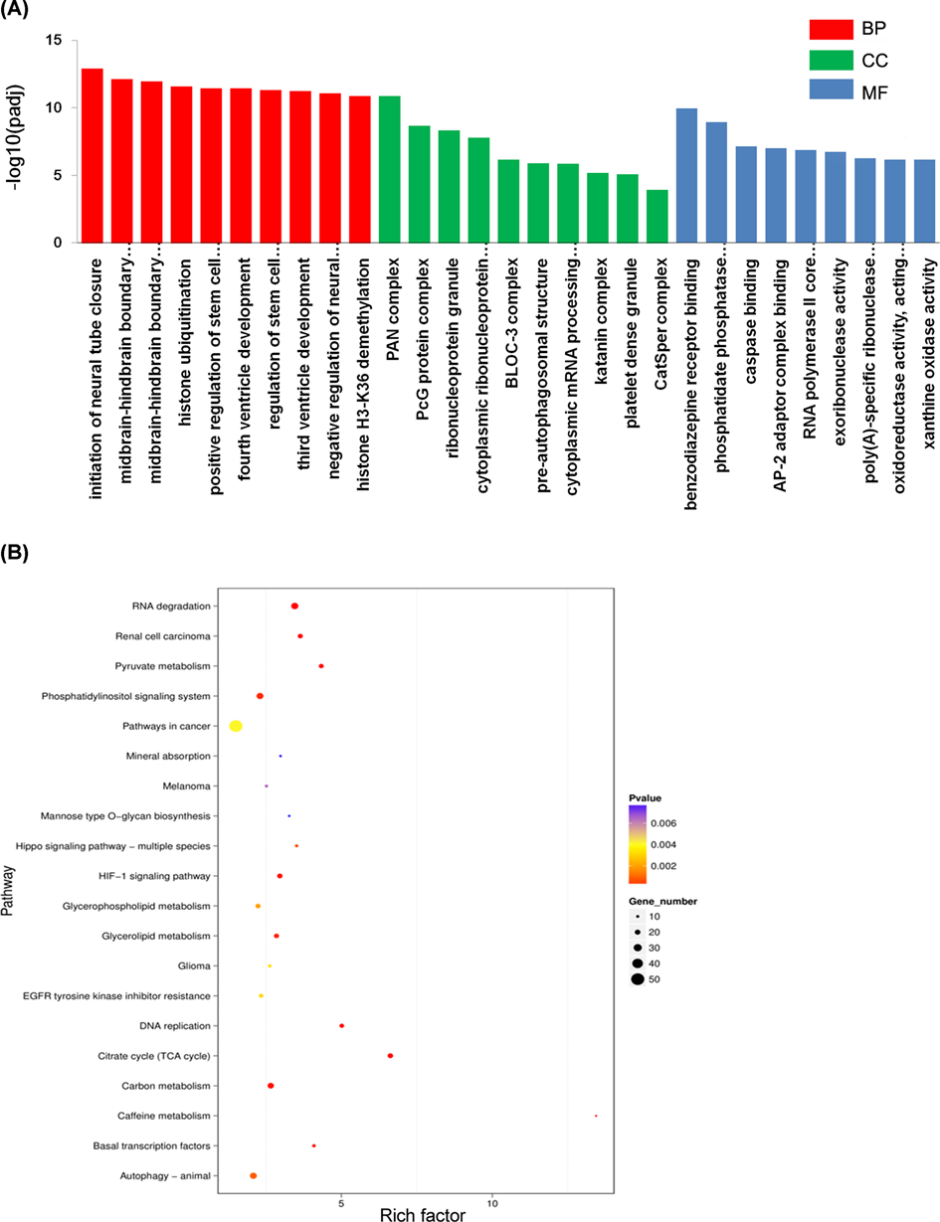
Enrichment analysis of novel DE miRNAs target genes (**A**) The results from novel DE miRNAs target genes were applied to GO analysis, with only showing the top ten significantly enriched GO terms in BP, CC, and MF (*P*<0.05). (**B**) KEGG pathway analysis of novel DE miRNAs target genes, with only showing the top 20 significantly enriched pathways (*P*<0.05). Rich factor = (target genes annotation in term/target genes with KEGG annotation)/(genes annotation in term/all genes with KEGG annotation).

A total of 1293 target genes from novel DE miRNAs were predicted using the miRanda and RNAhybrid software. GO analysis of novel DE miRNAs target genes showed enrichment of 3138 BP, 416 CC, and 550 MF, of which 817 BP (26.0%), 80 CC (19.2%), and 187 MF (34.0%) were significantly enriched (Supplementary Table S7). Figure 4A shows the top ten significantly enriched GO terms in the BP, CC, and MF categories. KEGG pathway analysis found 275 enriched pathways including RNA degradation, DNA replication, and pyruvate metabolism (Supplementary Table S8). The top 20 significantly enriched pathways are presented in Figure 4B.

### Validation of DE miRNAs

**Figure 5:**
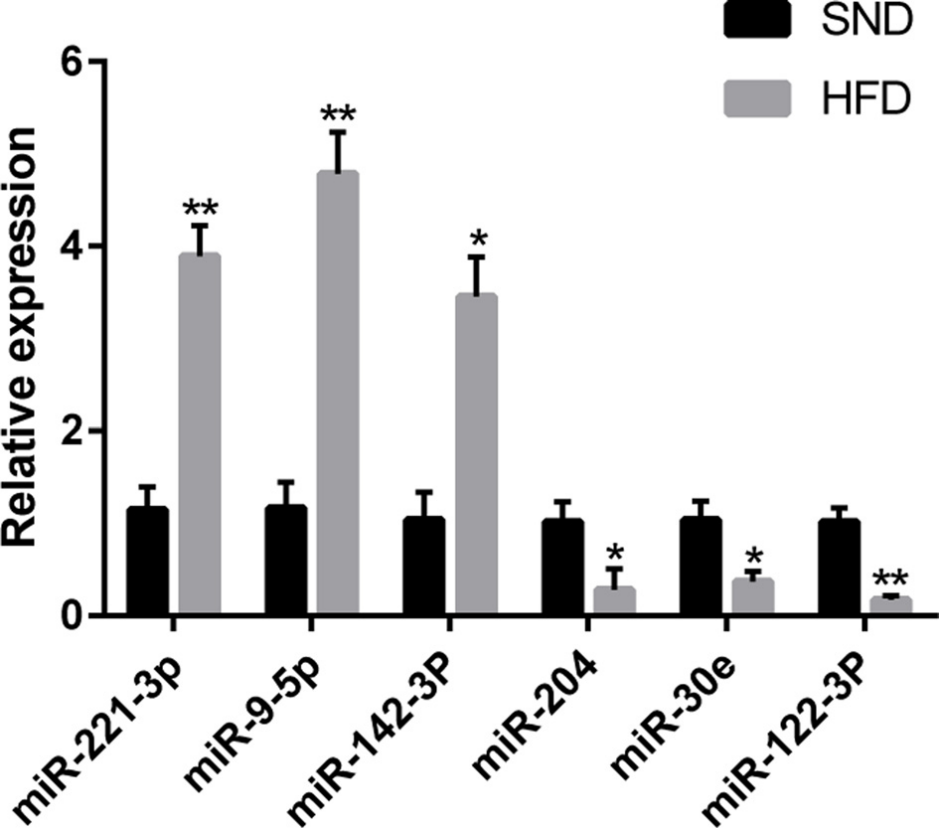
Validation of DE miRNAs Relative expression levels of the six randomly selected DE miRNAs by qRT-PCR. Data are expressed as means ± SEM; *n*=9 for each group; *P<0.05; **P<0.001.

We validated the sequencing results by qRT-PCR, using the total RNA from SND-1, SND-2, SND-3, HFD-1, HFD-2, HFD-3, and HFD-4. We randomly selected six DE miRNAs and examined their expression profiles. We confirmed an increased expression of miR-221-3p, miR-9-5p, and miR-142-3p at the RNA level in the HFD group, but miR-204, miR-30e, and miR-122-3p were down-regulated (Figure 5). These data demonstrated that the trend of miRNAs changes was consistent with the sequencing and qRT-PCR results.

## Discussion

The incidence of obesity is rapidly increasing in the world. Increasing study shows that miRNAs play a key role in the regulation of adipogenesis and obesity. Rabbits are emerging as a reliable and low-cost animal model for human obesity at a time when obesity and obesity-related problems are leading to increased morbidity and mortality [24]. WAT is the primary tissue affected by obesity [25]. In our experiment, we successfully constructed the obese rabbit model to investigate the miRNAs alterations underlying HFD by using a small RNA sequencing method. Moreover, six randomly selected DE miRNAs for qRT-PCR validation showed similar trends with small RNA sequencing. However, some limitations exist in the present study. For example, because the experimental objectives were animal specimens, the sample size of sequencing is relatively small. We did not use other public data and only used the qRT-PCR method to verify the results. Six randomly selected DE miRNAs cannot cover every aspect of DE miRNAs. Besides, we did not use different procedures to screen DE miRNAs for differential analyses. Thus, further verification of these DE miRNAs will be important to consider in the future.

In the present study, seven libraries (three SND and four HFD) were constructed for RNA-seq analysis of DE miRNAs. After processing and analyzing the raw reads, we obtained over 95% of clean reads, over 99.54% of Q20, and GC contents greater than 45.74% indicating that the cDNA libraries were high quality and the sequenced samples were highly reliable. The distribution of small RNA length of clean reads ranged from 18 to 30 nt, and the percentage of 22 nt reads was greater than any of the other reads. These results were consistent with research using backfat from male pigs and fat tail sheep [26,27]. However, other studies found that miRNAs length was influenced by age and tissue. The majority of small RNA-seq reads in the testis of 6 and 12 months old Xiang pigs (28–31 nt) was much greater than that of 2 and 3 months old Xiang pigs (21–23 nt), suggesting that the length of miRNAs was different in the testis of immature and mature animals [28]. The dominant size of the small RNA in the testes of breeder cocks was 26 nt, which was 4 nt longer than the length of miRNAs found in other chicken tissues [29]. Therefore, we hypothesized that differences in miRNAs length may be species, tissue, and development-specific.

Moreover, we discovered a total of 83 DE miRNAs (52 known DE miRNAs and 31 novel DE miRNAs). There was over a two-fold differential expression between miR-26a, miR-135a, miR-450b-5p, let-7f-1-3p, novel_mir190, novel_mir154, novel_mir546, novel_mir89, novel_mir208, novel_mir90, novel_mir450, and novel_mir425 from SND and HFD rabbits (*P*<0.001). Among these DE miRNAs, some miRNAs were found to be involved in the modulation of adipocyte growth and development in previous studies. For example, the miR-26 family positively regulates adipogenesis and is involved in the regulation of the function of thermogenic adipocytes [30]. Over-expression of let-7i-5p in murine scWAT limited the recruitment of brite adipocytes [31]. Interestingly, miR-9-5p has been reported to regulate white preadipocyte differentiation in culture by targeting the *leptin* gene [32]. Our findings provided the first evidence for the up-regulated expression of miR-9-5p in the perirenal adipose tissue of HFD-induced obese rabbits. However, the involvement of many miRNAs in regulating fat metabolism needs further *in vivo* and* in vitro* experiments is warranted to describe these miRNAs as a crucial role in the development of obesity.

miRNAs are a class of endogenous RNA that pair with the 3′ untranslated region (UTR) of target gene mRNA and regulate the expression of the target gene at the post-transcriptional level [9]. Extensive work revealed that miRNA is involved in the regulation of adipogenesis or obesity [33]. In our study, a total of 190 known DE miRNAs target genes were identified using the miRanda and RNAhybrid software and were further analyzed with GO and KEGG to obtain an overview of DE miRNAs and to further explore their function. The top ten significantly enriched terms in the BP, CC, and MF categories indicated the possible roles of the known DE miRNAs in regulating adipogenesis. Cytoskeletal remodeling and cell–cell interaction are a necessary step in the transformation of preadipocytes into mature adipocytes, and adipocyte development is dependent on α-tubulin acetylation [34,35]. Our data showed that regulation of cytoskeleton organization, intermediate filament cytoskeleton organization, intermediate filament-based process, α-tubulin binding, cell–substrate adherens junction, anchoring junction, cell–substrate junction, adherens junction, cell junction, intermediate filament, and focal adhesion GO terms were significantly enriched. Furthermore, other GO items related to adipogenesis were also significantly enriched, such as transforming growth factor beta receptor, pathway-specific cytoplasmic mediator activity, and lactate transmembrane transporter activity terms. Increased membrane lactic acid permeability is very important to adipocyte metabolism and is related to adipocyte thermogenesis, browning, and differentiation [36]. Adipogenesis is considered to be a dynamic process involving an elaborate network of genes, hormones, and pathways etc [11]. These factors are positive or negative regulators in adipogenesis. KEGG pathway analysis revealed that 14 pathways were significantly enriched, including the p53 signaling pathway, linoleic acid metabolism, and Wnt signaling pathway. The p53 gene is a tumor suppressor regulator that controls various cellular gene networks. An imbalance between energy intake and expenditure results in excess triacylglyceride accumulation in adipose tissue leading to an increase in oxidative stress and higher expression of tumor suppressor gene *p53 *[37,38]. Linoleic acid metabolism and Wnt signaling pathways are also associated with adipogenesis. As a family of fatty acids, linoleic acid was reported to participate in the regulation of energy metabolism, adipogenesis, inflammation, lipid metabolism, and apoptosis [39]. The Wnt family factors mediate cross-talk between adipose cells, ensuring that adipocyte growth and differentiation are coupled to energy storage demands [40]. Thus, the known DE miRNAs enriched in these pathways indicate that these DE miRNAs might be an important regulator in adipogenesis.

Moreover, GO analysis of the novel DE miRNAs target genes showed that histone ubiquitination, positive regulation of stem cell population maintenance, histone H3-K36 demethylation, cytoplasmic mRNA processing body, poly(A)-specific ribonuclease activity, oxidoreductase activity, acting on CH or CH_2_ groups, oxygen as acceptor, xanthine oxidase activity, oxidoreductase activity, acting on CH or CH_2_ groups, and NAD or NADP as acceptor were significantly enriched. KEGG analysis revealed that the target genes of the novel DE miRNAs were mainly involved in pyruvate metabolism, carbon metabolism, glycerolipid metabolism, glycerophospholipid metabolism, glyoxylate, and dicarboxylate metabolism, which are key process in the tricarboxylic acid (TCA) cycle or related life activities. The TCA cycle is a common metabolic pathway for carbohydrates, lipids, and amino acids in mitochondria, and provides precursors to other types of anabolism [41]. When the balance of energy and metabolism is destroyed, TCA cycle homeostasis will also be broken [42]. Thus, it is very important for the development of obesity to maintain the homeostasis of the TCA cycle. In conclusion, we think that DE novel miRNAs might regulate rabbit adipogenesis via the TCA cycle. However, their role in adipogenesis has not been fully studied in rabbits.

In conclusion, we applied a small RNA sequencing method to obtain comprehensive data of miRNAs in young female rabbits treated with a SND and HFD. We found that miRNAs were changed markedly and the p53 signaling pathway, linoleic acid metabolism pathway, glycerolipid metabolism pathway etc, may be the key pathways during adipogenesis. Our study provides further insights into the understanding of HFD-mediated obesity and may contribute to the prevention and treatment of obesity.

## Supplementary Material

Supplementary Tables S1-S8

## Data Availability

The data that support the findings of the present study are openly available in the NCBI database at https://trace.ncbi.nlm.nih.gov/Traces/sra/?study=SRP307855 (Bioproject ID: PRJNA704429).

## Abbreviations

BMI: body mass index
BP: biological process
CC: cellular component
DE: differentially expressed
EP: eppendorf
GO: Gene Ontology
HFD: high-fat diet
KEGG: Kyoto Encyclopedia of Genes and Genomes
MF: molecular function
miRNAs: microRNAs
qRT-PCR: quantitative real-time PCR
SND: standard normal diet
TCA: tricarboxylic acid
TG: triglyceride
WAT: white adipose tissue
